# Adverse Childhood Experiences and Nonsuicidal Self-Injury and Suicidality in Chinese Adolescents

**DOI:** 10.1001/jamanetworkopen.2024.52816

**Published:** 2024-12-30

**Authors:** Yitong He, Weiqing Jiang, Wanxin Wang, Qianyu Liu, Shuyi Peng, Lan Guo

**Affiliations:** 1 Department of Medical Statistics and Epidemiology, School of Public Health, Sun Yat-sen University, Guangzhou, China

## Abstract

**Importance:**

Studies investigating the role of supportive school environments in associations between adverse childhood experiences (ACEs) and nonsuicidal self-injury (NSSI) and suicidality among adolescents are lacking.

**Objective:**

To assess associations of ACEs with NSSI and suicidality among adolescents and examine the modifying role of supportive school environments in such associations.

**Design, Setting, and Participants:**

Using data from the 2021 School-Based Chinese Adolescents Health Survey, this cross-sectional study includes Chinese adolescents in grades 7 through 12 from 326 schools across 8 provinces in China. Statistical analysis was performed from March to October 2024.

**Exposure:**

ACEs, including individual indicators and cumulative numbers by total and categorized as threat-related and deprivation-related ACEs, were assessed.

**Main Outcome and Measures:**

NSSI, suicidal ideation, and suicide attempts were measured using validated questionnaires. Weighted Poisson regression models were used. The modifying interaction of supportive school environments was assessed using multiplicative interactions.

**Results:**

Among 95 549 adolescents included in this study (mean [SD] age, 14.9 [1.8] years; 47 617 males [49.8%]), 45 236 individuals (47.3%) had experienced at least 1 ACE. Compared with adolescents with no ACEs, those who experienced 4 or more ACEs had an increased risk of NSSI (prevalence ratio [PR], 1.31; 95% CI, 1.30-1.33) and suicidality (ideation: PR, 1.41; 95% CI, 1.39-1.42; attempts: PR, 1.25; 95% CI, 1.24-1.27) after adjusting for covariates, including supportive school environments. A dose-response association was observed between the number of ACEs and the risk of NSSI and suicidality (eg, NSSI: PR, 1.06; 95% CI, 1.05-1.06 for exposure to 1 vs 0 ACEs; PR, 1.10; 95% CI, 1.10-1.11 for exposure to 2 vs 0 ACEs). The highest PRs were found for associations of threat-related ACEs (eg, physical abuse, emotional abuse, and bullying) with NSSI and suicidality (eg, exposure to ≥2 vs 0 threat-related ACEs: PR, 1.28; 95% CI, 1.27-1.29 for NSSI; PR, 1.33; 95% CI 1.32-1.34 for ideation; PR, 1.18; 95% CI, 1.17-1.19 for attempts). All individual ACE indicators were positively associated with NSSI and suicidality, with particularly high PRs for emotional abuse (eg, PR, 1.26; 95% CI, 1.24-1.27 for NSSI). Additionally, supportive school environments significantly modified associations of ACEs with NSSI and suicidality (eg, interaction ratio for NSSI, 0.81; 95% CI, 0.76-0.88).

**Conclusion and relevance:**

In this study, exposure to ACEs was associated with an increased risk of NSSI and suicidality, with particularly high increases for threat-related ACEs, independent of the interaction of supportive school environments, but supportive school environments could modify such detrimental outcomes. These findings underscore the importance of enhancing school environments to prevent NSSI and suicidality among adolescents who have experienced ACEs.

## Key Points

**Question:**

Are adverse childhood experiences (ACEs) associated with nonsuicidal self-injury (NSSI) or suicidality among Chinese adolescents, and does a supportive school environment modify such associations?

**Findings:**

In this cross-sectional study involving 95 549 adolescents in China, exposure to ACEs was associated with an increased risk of NSSI and suicidality, with particularly high increases in risk for threat-related ACEs. Associations of ACEs with NSSI and suicidality were modified by supportive school environments.

**Meaning:**

These findings suggest the potential benefits of enhancing school environments in preventing NSSI and suicidality among individuals who have experienced ACEs.

## Teaser

This cross-sectional study investigates the association of adverse childhood experiences with nonsuicidal self injury and suicidality among Chinese adolescents.

## Introduction

Nonsuicidal self-injury (NSSI) refers to the direct and deliberate destruction of body tissue without suicidal intent, which is distinct from suicidality (eg, suicidal ideation [SI] and suicide attempt [SA]) in both purpose and frequency. NSSI, SI, and SA are persistent public health issues among adolescents worldwide,^1,2^ with prevalence possibly increasing during the COVID-19 pandemic. The impact of NSSI and suicidality on adolescents can be profound, often manifesting as various subclinical psychosocial difficulties that are associated with large increases in odds of future suicidal behaviors.^3^ Therefore, identifying modifiable factors associated with NSSI and suicidality is crucial for planning public health interventions and promoting adolescent psychosocial health.

Although previous research has identified several risk factors associated with NSSI and suicidality, such as interpersonal relationships, academic stress, and mental illness,^4^ there is also evidence suggesting an association between adverse childhood experiences (ACEs) and NSSI and suicidality.^5,6^ ACEs encompass a broad spectrum of potentially stressful encounters during childhood,^7^ with estimates indicating that 27% to 70% of Chinese adolescents are exposed to at least 1 ACE.^8,9^ However, most previous studies have been conducted on adults using retrospectively collected information on ACEs, primarily focusing on NSSI or suicidality in adulthood.^10,11^ In contrast to the relatively well-studied ACEs among Western populations, only a few investigations, with limited sample sizes or restricted geographic areas, have explored the association of ACEs with NSSI and suicidality among Chinese adolescents.^12,13^ Moreover, aside from long-term outcomes, the role of ACEs in the sensitive neurological and brain structural development stage of adolescence^14,15^ has been less studied.

Additionally, previous research often focused on the conventional set of ACEs from the Centers for Disease Control and Prevention (CDC)-Kaiser Permanente ACE study,^16,17^ which cover abuse, neglect, and household dysfunction. However, recent studies have highlighted that conventional ACEs may not adequately reflect perceived childhood adversity. An expanded set of ACEs, including school bullying and community violence, was outlined in the ACE International Questionnaire (ACE-IQ) developed by the World Health Organization^18^ and has also been reported to be prevalent and associated with NSSI and suicidality.^19,20^ Nevertheless, previous studies typically conceptualized ACEs using the cumulative risk model, which suggested that all types of ACEs were associated with NSSI and suicidality through the same underlying mechanism.^5,6,12^ An alternative approach, the dimensional model of adversity and psychopathology, was further proposed to differentiate threat-related and deprivation-related ACEs because these 2 dimensions were considered to be associated with emotion regulation and cognitive control in adolescence.^21^

During the past decade, emerging studies have striven to identify potential effect modifiers that may interact in associations between ACEs and adverse outcomes.^22,23^ These may help develop tailored interventions to mitigate the negative outcomes associated with ACEs among adolescents. School is the place where adolescents spend most of their time, and it plays a critical role in promoting student health and development. Current evidence suggests that a positive and supportive school environment can promote adolescent mental health^24,25^ and is negatively associated with NSSI.^26^ Moreover, a cross-sectional study in the US^27^ suggested that supportive school environments may buffer the association between ACEs and suicidality. Nevertheless, knowledge regarding the modifying role of supportive school environments in the associations of expanded ACEs with NSSI and suicidality is limited.

In this nationwide, large-scale cross-sectional study, we aimed to comprehensively estimate associations of ACEs (individual indicator and cumulative numbers by total and categorized as threat-related and deprivation-related ACEs) with NSSI and suicidality among Chinese adolescents. The modifying interaction of supportive school environments in investigated associations was also evaluated.

## Methods

### Study Design and Participants

This cross-sectional study used data from the 2021 School-Based Chinese Adolescents Health Survey (SCAHS), an ongoing survey of health-related behaviors among Chinese adolescents in grades 7 through 12.^28^ The 2021 SCAHS used a multistage, stratified cluster random sampling method; detailed data-collection procedures have been described previously.^29^ A total of 98 192 students from 326 high schools across 8 provinces of China participated in this survey, yielding an overall response rate of 97.3%. Self-reported questionnaires were administered in classrooms.^30^ For this analysis, we excluded 2643 participants with missing data on ACEs, NSSI, and suicidality, resulting in a final sample of 95 549 participants (age range, 11-21 years). The detailed process of participant selection is listed in eMethods and eFigure 1 in Supplement 1. Participation in the SCAHS was voluntary, with written informed consent obtained from all participants and 1 of their legal guardians. Ethical approval was granted by the institutional review board of Sun Yat-sen University School of Public Health. This study adhered to the Strengthening the Reporting of Observational Studies in Epidemiology (STROBE) reporting guideline.

### Adverse Childhood Experiences

Based on the CDC-Kaiser Permanente ACE study^16,17^ and the ACE-IQ,^18^ we collected data on 15 conventional and expanded forms of ACEs through self-reported questionnaires. These ACEs were further categorized into threat-related and deprivation-related dimensions. Details are presented in the eMethods in Supplement 1. Each ACE indicator was dichotomized and summed to generate a cumulative ACE score ranging from 0 to 15. Participants were further categorized into 5 groups based on cumulative ACE score: 0, 1, 2, 3, and 4 or higher.^29^ Additionally, cumulative scores for threat-related and deprivation-related ACEs were generated, and participants were grouped into 3 categories based on these scores: 0, 1, and 2 or higher for both threat-related and deprivation-related ACEs.^31^

### Nonsuicidal Self-Injury

NSSI was assessed using the Chinese version of the Functional Assessment of Self-Mutilation.^32^ Participants were asked, “In the past 12 months, have you ever harmed yourself deliberately without intending to take your life?” We provided 8 different forms of NSSI, including head banging, hair pulling, pinching, hitting, burning, biting, cutting, and scratching. For participants who reported engaging in NSSI, the frequency of NSSI was assessed and a total NSSI frequency was calculated. In this study, NSSI was dichotomized based on total frequency, with participants reporting 3 or more incidents categorized as “yes” and those with fewer than 3 incidents categorized as “no.”^12,33,34,35^

### Suicidal Ideation and Suicide Attempt

SI was defined as responding, “1 or more times,” to the question, “During the past 12 months, how many times did you seriously consider attempting suicide?” SA was assessed by the question “During the past 12 months, how many times did you attempt suicide?” and asked that the student rate on a scale of zero or once or more.^36^

### Supportive School Environment

The supportive school environment was evaluated using 3 questions: (1) “How would you describe your relationship with your classmates?”, (2) “How would you describe your relationship with the teachers in your class?”, and (3) “At school, how many of your close friends frequently make mistakes?”^37^ Responses to each question were dichotomized, with 0 indicating a poor situation and 1 indicating a good situation. A total score ranging from 0 to 3 was then calculated, with higher scores suggesting more supportive school environments.

### Covariates

Covariates assessed included age, sex (male and female), current drinking status (yes and no), current smoking status (yes and no), household socioeconomic status (>mean, mean, and <mean), academic pressure (none, moderate, and severe), and depressive symptoms (measured using the 20-item Center for Epidemiologic Studies Depression Scale, as detailed in the eMethods in Supplement 1). To minimize bias in estimating associations of ACEs and supportive school environments with NSSI and suicidality, a directed acyclic graph was used to select the appropriate set of covariates for adjustment (eFigure 2 in Supplement 1).^38^

### Statistical Analysis

Data are presented as means and SDs for continuous variables and frequencies with percentages for categorical variables. Characteristics were compared across NSSI, SI, and SA using analysis of variance for continuous variables and χ^2^ tests for categorical variables, as appropriate. Prevalence estimates and corresponding 95% CIs for NSSI, SI, and SA were calculated and weighted for the complex sampling design and nonresponse.^39^ Weighted Poisson regression models that accounted for the stratified cluster survey design were performed to test associations of ACEs and supportive school environments with NSSI, SI, and SA, and prevalence ratios (PRs) with 95% CIs were estimated (details are presented in the eMethods in Supplement 1). ACE exposure was treated as a continuous variable, binary variable, and categorical variable, and individual ACEs were assessed. Moreover, multiplicative interaction analyses were conducted to assess the modifying interaction of supportive school environments with associations between ACEs and NSSI, SI, and SA. An interaction product term between ACEs and supportive school environments was included in models. Multiplicative interaction was measured by the interaction ratio, calculated as PR_interaction term_/(PR_ACEs_ × PR_supportive school environment_).^40,41^ A positive multiplicative interaction was indicated by an interaction ratio greater than 1, a negative interaction by a ratio less than 1, and no interaction by a ratio equal to 1.^42^ To explore potential variations across subgroups, we conducted subgroup analyses stratified by sex (males and females).

We conducted additional sensitivity analyses to validate the robustness of our main findings. First, we used an alternative cutoff for NSSI, categorizing individuals who reported 5 or more ACE incidents as “yes” and those with fewer than 5 ACE incidents as “no.”^43^ Second, we additionally adjusted for depressive symptoms, which were not initially included in the main analysis owing to their potential role as a mediator in associations of ACEs with NSSI and suicidality.^44^ All statistical analyses were performed using R statistical software version 4.2.2 (R Foundation for Statistical Computing), with a two-sided *P*-value < .05 considered statistically significant. Analyses were performed from March to October 2024.

## Results

Among 95 549 participants (47 617 male [49.8%]; mean [SD] age, 14.9 [1.8] years), 45 236 participants (47.3%) had been exposed to at least 1 of 15 ACEs. Table 1 presents characteristics of the study population. The mean (SD) score of supportive school environments was 1.7 (0.9). As shown in eTable 1 in Supplement 1, and the prevalence was 15.0% (95% CI, 14.8%-15.3%) for NSSI, 18.7% (95% CI, 18.4%-19.0%) for SI, and 5.6% (95% CI, 5.5%-5.8%) for SA.

**Table 1.** Characteristics of Participants

Characteristic	Participants, No. (%)			
	Overall (N = 95 549)	NSSI (n = 14 481)	Suicidal ideation (n = 17 866)	Suicide attempt (n = 5315)
Age, mean (SD), y	14.93 (1.76)	14.75 (1.69)	14.77 (1.69)	14.56 (1.63)
Sex				
Male	47 617 (49.8)	5357 (37.0)	6253 (35.0)	1598 (30.1)
Female	47 932 (50.2)	9123 (63.0)	11 613 (65.0)	3717 (69.9)
HSS				
>Mean	24 864 (26.0)	3230 (22.3)	3862 (21.6)	1225 (23.0)
Mean	56 827 (59.5)	8506 (58.7)	10 598 (59.3)	2989 (56.2)
<Mean	13 584 (14.2)	2713 (18.7)	3367 (18.8)	1087 (20.5)
Missing	274 (0.3)	31 (0.2)	39 (0.2)	14 (0.3)
Current smoking status				
No	5299 (5.5)	1266 (8.7)	1628 (9.1)	752 (14.1)
Yes	90 250 (94.5)	13 214 (91.3)	16 238 (90.9)	4563 (85.9)
Current drinking status				
No	4826 (5.1)	1195 (8.3)	1522 (8.5)	703 (13.2)
Yes	90 723 (94.9)	13 285 (91.7)	16 344 (91.5)	4612 (86.8)
Academic pressure				
None	20 036 (21.0)	1698 (11.7)	2073 (11.6)	644 (12.1)
Moderate	43 441 (45.5)	5421 (37.4)	6570 (36.8)	1848 (34.8)
Severe	31 846 (33.3)	7331 (50.6)	9185 (51.4)	2810 (52.9)
Missing	226 (0.2)	30 (0.2),	38 (0.2)	13 (0.2)
Supportive school environment, mean (SD)	1.97 (0.91)	1.64 (0.96)	1.63 (0.96)	1.50 (0.97)
ACEs, No./participant, mean (SD)	0.95 (1.37)	1.90 (1.93)	1.96 (1.90)	2.62 (2.16)
ACEs status				
No	50 313 (52.7)	4128 (28.5)	4631 (25.9)	906 (17.0)
Yes	45 236 (47.3)	10 352 (71.5)	13 235 (74.1)	4409 (83.0)
No. of ACEs				
0	50 313 (52.7)	4128 (28.5)	4631 (25.9)	906 (17.0)
1	21 921 (22.9)	3369 (23.3)	4232 (23.7)	1022 (19.2)
2	12 304 (12.9)	2591 (17.9)	3366 (18.8)	982 (18.5)
3	5563 (5.8)	1814 (12.5)	2328 (13.0)	835 (15.7)
≥4	5448 (5.7)	2578 (17.8)	3309 (18.5)	1570 (29.5)
No. of threat-related ACEs				
0	69 953 (73.2)	6424 (44.4)	7755 (43.4)	1744 (32.8)
1	16 314 (17.1)	3969 (27.4)	5029 (28.1)	1429 (26.9)
≥2	9282 (9.7)	4087 (28.2)	5082 (28.4)	2142 (40.3)
No. of deprivation-related ACEs				
0	62 206 (65.1)	7286 (50.3)	8409 (47.1)	1947 (36.6)
1	20 628 (21.6)	3816 (26.4)	4928 (27.6)	1460 (27.5)
≥2	12 715 (13.3)	3378 (23.3)	4529 (25.3)	1908 (35.9)

Abbreviations: ACE, adverse childhood experience; HSS, household socioeconomic status; NSSI, nonsuicidal self-injury.

A higher number of ACEs was associated with increased risks of NSSI and suicidality (eg, PR per 1-unit increase in ACEs, 1.05; 95% CI, 1.05-1.06 for NSSI in the fully adjusted model 2). A dose-response association was observed between cumulative ACE exposure and NSSI and suicidality, even in the fully adjusted model 2 (eg, NSSI: PR, 1.06; 95% CI, 1.05-1.06 for exposure to 1 vs 0 ACEs; PR, 1.10; 95% CI, 1.10-1.11 for exposure to 2 vs 0 ACEs). For 4 or more ACEs compared with 0 ACEs in model 2, PRs were 1.31 (95% CI, 1.30-1.33) for NSSI, 1.41 (95% CI, 1.39-1.42) for SI, and 1.25 (95% CI, 1.24-1.27) for SA (Table 2). Furthermore, threat-related and deprivation-related ACEs were positively associated with NSSI and suicidality, even in the fully adjusted model 2 (eg, ≥2 vs 0 threat-related ACEs: PR, 1.28; 95% CI, 1.27-1.29 for NSSI; PR, 1.33; 95% CI 1.32-1.34 for SI; PR, 1.18; 95% CI, 1.17-1.19 for SA). Similar results were observed across male and female subgroups (eTables 2-3 in Supplement 1).

**Table 2.** Associations of Cumulative ACEs With NSSI and Suicidality

ACEs	PR (95% CI)						
	NSSI		Suicidal ideation		Suicide attempt		
	Model 1^a^	Model 2^b^	Model 1^a^	Model 2^b^	Model 1^a^		Model 2^b^
Cumulative ACEs, per 1-unit increase	1.31 (1.30-1.32)	1.05 (1.05-1.06)	1.32 (1.31-1.33)	1.07 (1.07-1.07)	1.46 (1.44-1.48)	1.04 (1.04-1.04)	
Any ACE, No							
0	1 [Reference]	1 [Reference]	1 [Reference]	1 [Reference]	1 [Reference]	1 [Reference]	
≥1	2.56 (2.46-2.66)	1.11 (1.11-1.12)	2.87 (2.77-2.98)	1.15 (1.15-1.16)	4.81 (4.43-5.23)	1.07 (1.06-1.07)	
No. of ACEs							
0	1 [Reference]	1 [Reference]	1 [Reference]	1 [Reference]	1 [Reference]	1 [Reference]	
1	1.86 (1.77-1.95)	1.06 (1.05-1.06)	2.05 (1.96-2.14)	1.08 (1.08-1.09)	2.64 (2.39-2.93)	1.03 (1.02-1.03)	
2	2.47 (2.34-2.60)	1.10 (1.10-1.11)	2.83 (2.70-2.96)	1.15 (1.14-1.16)	4.34 (3.92-4.81)	1.06 (1.05-1.06)	
3	3.63 (3.43-3.84)	1.20 (1.18-1.21)	4.06 (3.87-4.26)	1.26 (1.25-1.28)	7.41 (6.66-8.24)	1.12 (1.11-1.13)	
≥4	4.83 (4.59-5.07)	1.31 (1.30-1.33)	5.42 (5.19-5.66)	1.41 (1.39-1.42)	13.07 (11.88-14.38)	1.25 (1.24-1.27)	
Cumulative threat-related ACEs, per 1-unit increase	1.51 (1.49-1.53)	1.09 (1.09-1.10)	1.50 (1.48-1.51)	1.11 (1.11-1.11)	1.70 (1.67-1.73)	1.07 (1.06-1.07)	
No. of threat-related ACEs							
0	1 [Reference]	1 [Reference]	1 [Reference]	1 (Reference	1 [Reference]	1 [Reference]	
1	2.48 (2.37-2.58)	1.12 (1.11-1.13)	2.53 (2.44-2.62)	1.15 (1.14-1.16)	3.25 (3.00-3.52)	1.05 (1.05-1.06)	
≥2	4.07 (3.91-4.24)	1.28 (1.27-1.29)	4.06 (3.93-4.21)	1.33 (1.32-1.34)	7.46 (6.92-8.04)	1.18 (1.17-1.19)	
Cumulative deprivation-related ACEs, per 1-unit increase	1.34 (1.32-1.36)	1.04 (1.04-1.05)	1.42 (1.41-1.44)	1.07 (1.07-1.07)	1.71 (1.67-1.76)	1.04 (1.04-1.05)	
No. of deprivation-related ACEs							
0	1 [Reference]	1 [Reference]	1 [Reference]	1 [Reference]	1 [Reference]	1 [Reference]	
1	1.50 (1.44-1.56)	1.05 (1.04-1.05)	1.66 (1.60-1.72)	1.07 (1.07-1.08)	2.10 (1.94-2.27)	1.03 (1.03-1.04)	
≥2	2.01 (1.92-2.09)	1.10 (1.09-1.11)	2.35 (2.26-2.43)	1.16 (1.15-1.17)	4.03 (3.74-4.34)	1.10 (1.09-1.11)	

Abbreviations: ACE, adverse childhood experience; NSSI, nonsuicidal self-injury; PR, prevalence ratio.

^a^ Model 1 was adjusted for age, sex, current drinking status, current smoking status, household socioeconomic status, and academic pressure.

^b^ Model 2 was adjusted for the variables in model 1, plus supportive school environments.

Most ACE subtypes were positively associated with an increased risk of NSSI and suicidality, even in the fully adjusted model 2. Specifically, the associations with the highest PRs were found for emotional abuse (eg, PR, 1.26; 95% CI, 1.24-1.27 for NSSI) (Table 3). Similar findings were observed in male and female subgroups (eTables 4-5 in Supplement 1).

**Table 3.** Associations of Each ACE Subtype With NSSI and Suicidality

ACE subtype	PR (95% CI)					
	NSSI		Suicidal ideation		Suicide attempt	
	Model 1^a^	Model 2^b^	Model 1^a^	Model 2^b^	Model 1^a^	Model 2^b^
**Threat-related ACEs**						
Physical abuse						
No	1 [Reference]	1 [Reference]	1 [Reference]	1 [Reference]	1 [Reference]	1 [Reference]
Yes	1.72 (1.65-1.78)	1.25 (1.23-1.27)	2.81 (2.69-2.93)	1.30 (1.28-1.32)	4.40 (4.04-4.79)	1.21 (1.19-1.23)
Sex abuse						
No	1 [Reference]	1 [Reference]	1 [Reference]	1 [Reference]	1 [Reference]	1 [Reference]
Yes	2.59 (2.44-2.75)	1.21 (1.19-1.23)	2.31 (2.19-2.44)	1.21 (1.19-1.23)	3.52 (3.19-3.87)	1.15 (1.13-1.17)
Emotional abuse						
No	1 [Reference]	1 [Reference]	1 [Reference]	1 [Reference]	1 [Reference]	1 [Reference]
Yes	2.86 (2.74-2.97)	1.26 (1.24-1.27)	2.98 (2.89-3.08)	1.33 (1.32-1.34)	4.99 (4.67-5.33)	1.22 (1.21-1.23)
Household substance abuse						
No	1 [Reference]	1 [Reference]	1 [Reference]	1 [Reference]	1 [Reference]	1 [Reference]
Yes	2.37 (2.17-2.59)	1.19 (1.16-1.22)	2.36 (2.19-2.53)	1.23 (1.21-1.26)	3.12 (2.69-3.62)	1.14 (1.11-1.17)
Witness of community violence						
No	1 [Reference]	1 [Reference]	1 [Reference]	1 [Reference]	1 [Reference]	1 [Reference]
Yes	2.18 (2.08-2.28)	1.15 (1.13-1.16)	1.97 (1.89-2.05)	1.15 (1.13-1.16)	2.50 (2.31-2.72)	1.08 (1.07-1.09)
Household domestic violence						
No	1 [Reference]	1 [Reference]	1 [Reference]	1 [Reference]	1 [Reference]	1 [Reference]
Yes	2.12 (2.03-2.21)	1.14 (1.13-1.15)	2.26 (2.18-2.34)	1.19 (1.18-1.2)	2.89 (2.69-3.10)	1.09 (1.09-1.10)
Sex discrimination						
No	1 [Reference]	1 [Reference]	1 [Reference]	1 [Reference]	1 [Reference]	1 [Reference]
Yes	2.22 (2.11-2.33)	1.18 (1.17-1.20)	2.11 (2.02-2.20)	1.20 (1.19-1.22)	2.67 (2.44-2.91)	1.11 (1.10-1.13)
Bullying						
No	1 [Reference]	1 [Reference]	1 [Reference]	1 [Reference]	1 [Reference]	1 [Reference]
Yes	2.51 (2.42-2.60)	1.16 (1.15-1.17)	2.37 (2.29-2.44)	1.18 (1.17-1.19)	3.26 (3.06-3.49)	1.10 (1.09-1.10)
**Deprivation-related ACEs**						
Physical neglect						
No	1 [Reference]	1 [Reference]	1 [Reference]	1 [Reference]	1 [Reference]	1 [Reference]
Yes	1.47 (1.42-1.53)	1.05 (1.04-1.06)	1.65 (1.60-1.71)	1.08 (1.08-1.09)	2.42 (2.27-2.58)	1.06 (1.05-1.06)
Emotional neglect						
No	1 [Reference]	1 [Reference]	1 [Reference]	1 [Reference]	1 [Reference]	1 [Reference]
Yes	1.80 (1.73-1.88)	1.09 (1.08-1.10)	2.05 (1.98-2.11)	1.14 (1.14-1.15)	3.18 (2.98-3.39)	1.10 (1.09-1.10)
Parental divorce						
No	1 [Reference]	1 [Reference]	1 [Reference]	1 [Reference]	1 [Reference]	1 [Reference]
Yes	1.35 (1.29-1.41)	1.04 (1.04-1.05)	1.52 (1.47-1.58)	1.08 (1.07-1.09)	1.77 (1.64-1.90)	1.04 (1.03-1.05)
Household criminality						
No	1 [Reference]	1 [Reference]	1 [Reference]	1 [Reference]	1 [Reference]	1 [Reference]
Yes	1.46 (1.31-1.63)	1.06 (1.04-1.08)	1.8 (1.66-1.95)	1.13 (1.11-1.15)	2.06 (1.76-2.42)	1.06 (1.04-1.08)
Household mental illness						
No	1 [Reference]	1 [Reference]	1 [Reference]	1 [Reference]	1 [Reference]	1 [Reference]
Yes	2.42 (2.24-2.63)	1.20 (1.17-1.23)	2.18 (2.03-2.34)	1.20 (1.18-1.23)	3.19 (2.79-3.64)	1.14 (1.11-1.17)
Family financial problems						
No	1 [Reference]	1 [Reference]	1 [Reference]	1 [Reference]	1 [Reference]	1 [Reference]
Yes	1.68 (1.58-1.79)	1.09 (1.07-1.10)	1.62 (1.54-1.71)	1.10 (1.08-1.11)	1.74 (1.56-1.94)	1.04 (1.03-1.05)
Parental death						
No	1 [Reference]	1 [Reference]	1 [Reference]	1 [Reference]	1 [Reference]	1 [Reference]
Yes	1.16 (1.05-1.29)	1.02 (1.01-1.04)	1.16 (1.06-1.27)	1.03 (1.01-1.05)	1.17 (0.98-1.40)	1.01 (1.00-1.02)

Abbreviations: ACE, adverse childhood experience; NSSI, nonsuicidal self-injury; PR, prevalence ratio.

^a^ Model 1 was adjusted for age, sex, current drinking status, current smoking status, household socioeconomic status, and academic pressure.

^b^ Model 2 was adjusted for the variables in model 1, plus supportive school environments.

A supportive school environment was negatively associated with NSSI and suicidality, even in the fully adjusted model 2. For example, a 1-unit increase in the supportive school environment score was associated with a lower prevalence of NSSI (PR, 0.75; 95% CI, 0.73-0.76) (Table 4). Similar results were observed across male and female subgroups (eTables 6-7 in Supplement 1).

**Table 4.** Associations of Supportive School Environment With NSSI and Suicidality

Supportive school environment outcome^a^	PR per 1-unit increase (95% CI)	
	Model 1^b^	Model 2^c^
NSSI	0.70 (0.68-0.71)	0.75 (0.73-0.76)
Suicidal ideation	0.69 (0.68-0.70)	0.74 (0.73-0.75)
Suicide attempt	0.60 (0.59-0.62)	0.66 (0.64-0.68)

Abbreviations: NSSI, nonsuicidal self-injury; PR, prevalence ratio.

^a^ The supportive school environment is a continuous variable, which was evaluated using 3 questions. The total score ranges from 0 to 3, with higher scores suggesting more supportive school environments.

^b^ Model 1 was unadjusted.

^c^ Model 2 was adjusted for age, sex, current drinking status, current smoking status, household socioeconomic status, and academic pressure.

Moreover, we observed multiplicative interactions between ACEs and supportive school environments in the association with NSSI and suicidality, even in the fully adjusted model 2 (eg, interaction ratio for NSSI, 0.81, 95% CI, 0.76-0.88) (Table 5). Similar results were observed across male and female subgroups (eTables 8-9 in Supplement 1).

**Table 5.** Interactions Between ACEs and Supportive School Environment

Outcome	Model 1^a^	Model 2^b^
**NSSI**		
PR per 1-unit increase (95% CI)		
ACE (≥1 vs 0)	2.10 (1.93-2.28)	1.89 (1.74-2.05)
Supportive school environment^c^	0.71 (0.69-0.74)	0.74 (0.71-0.76)
ACE × supportive school environment	1.12 (1.07-1.17)	1.13 (1.09-1.18)
Interaction ratio (95% C)^d^	0.75 (0.73-0.80)	0.81 (0.76-0.88)
**Suicidal ideation**		
PR per 1-unit increase (95% CI)		
ACE (≥1 vs 0)	2.32 (2.15-2.51)	2.07 (1.92-2.23)
Supportive school environment^c^	0.71 (0.69-0.74)	0.73 (0.71-0.76)
ACE × supportive school environment	1.13 (1.09-1.17)	1.15 (1.11-1.19)
Interaction ratio (95% CI)^d^	0.67 (0.62-0.73)	0.78 (0.70-0.81)
**Suicide attempt**		
PR per 1-unit increase (95% C)		
ACE (≥1 vs 0)	4.63 (3.89-5.52)	3.85 (3.24-4.57)
Supportive school environment^c^	0.70 (0.64-0.75)	0.70 (0.65-0.76)
ACE × supportive school environment	1.02 (0.94-1.12)	1.06 (0.98-1.16)
Interaction ratio (95% CI)^d^	0.31 (0.27-0.37)	0.39 (0.33-0.47)

Abbreviations: ACE, adverse childhood experience; NSSI, nonsuicidal self-injury; PR, prevalence ratio.

^a^ Model 1 was unadjusted.

^b^ Model 2 was adjusted for age, sex, current drinking status, current smoking status, household socioeconomic status, and academic pressure.

^c^ The supportive school environment is a continuous variable, which was evaluated using 3 questions. The total score ranges from 0 to 3, with higher scores suggesting more supportive school environments.

^d^ Calculated as PR_ACE × supportive school environment_/(PR_ACEs_ × PR_supportive school environment_).

As shown in eTables 10 to 12 in Supplement 1, results remained consistent with main findings when using an NSSI threshold of 5 or more times. Additionally, after further adjusting for depressive symptoms, findings were generally similar to main results (eTables 13-16 in Supplement 1).

## Discussion

This cross-sectional study found that exposure to ACEs, particularly threat-related ACEs, was independently associated with NSSI, SI, and SA among Chinese adolescents. Associations exhibited a dose-response pattern. Moreover, our study found that a supportive school environment was negatively associated with NSSI and suicidality, and it modified associations of ACEs with NSSI, SI, and SA.

In line with previous studies,^33,45^ we found that cumulative ACEs and each subtype of ACE were positively associated with NSSI, SI, and SA among adolescents. Similarly, a cross-sectional study conducted in the US^27^ found that compared with youths with no ACE exposure, middle school youths with 3 or more ACEs had a prevalence of suicidal behaviors that was 5.50 to 8.03 times higher, while high school youths with 3 or more ACEs had a prevalence that was 3.63 to 4.35 times higher. Although associations of ACEs with NSSI and suicidality have been consistently reported across various cultural contexts,^13,46,47^ limited research has explored the independent role of threat-related and deprivation-related ACEs in these outcomes in general adolescent populations. A previous cross-sectional study in a province of China^13^ reported that threat-related ACEs had associations with the greatest increases in odds of psychosocial difficulties. Another longitudinal study^48^ found a more pronounced increase in depression trajectories among individuals with threat-related ACEs than among individuals with other types of ACEs. Our study extends these findings by highlighting the specific association of threat-related ACEs, particularly emotional abuse, with NSSI, SI, and SA among adolescents. Although these findings should be validated through future cohort studies and randomized clinical trials, they highlight the potential for early identification and targeted interventions for adolescents exposed to threat-related ACEs, which may help reduce the risk of NSSI and suicidality.

Furthermore, our study found that certain types of ACEs under an expanded definition (eg, bullying) were associated with greater increases in risk of NSSI and suicidality than those observed with some conventional ACEs (eg, physical neglect). These findings suggest that the associated outcomes and significance of certain additional ACEs are substantial and warrant attention. A related study^31^ highlighted that if only conventional ACEs were considered, ACE exposure would have been underreported in 14.2% of included participants. Furthermore, the conventional ACE framework was originally developed based on a predominantly White, educated sample.^17^ Given that the perception and types of adversities may vary across different populations, there remains a need to develop culturally relevant ACE measurements. However, the exact underlying mechanisms in the association of ACEs with NSSI and suicidality remain unclear. One possible explanation is that early life adversity, especially including threat-related ACEs, may disrupt the development of brain regions involved in coping, self-regulation, and emotional management. Such disruption can result in emotional dysregulation, leading to maladaptive behaviors, including NSSI and suicidality, as a means of managing negative emotions.^49^

Previous research has established associations between social support and adolescent NSSI and suicidality.^33,43,50^ Our study extended this understanding by highlighting the association of support from school with risk of NSSI and suicidality among adolescents. Consistent with previous studies,^51,52^ our findings suggest that supportive school environments may be associated with reduced risk of adolescents engaging in NSSI and suicidality. Moreover, our study found that supportive school environments mitigated associations of ACEs with NSSI and suicidality, particularly among individuals with the highest level of ACE exposure. Interaction analysis suggest that supportive school environments could buffer the association of ACEs with these outcomes. These findings highlight the crucial role of the school environment in promoting adolescent mental health, which aligns with existing evidence.^25,53^ For instance, a 2021 cross-sectional study in the US^27^ found that school connectedness, an indicator of supportive school environments, moderated the association between ACEs and suicidal behaviors (eg, suicidal planning and suicide attempts). Similarly, a quasiexperimental study in the US^54^ revealed that individuals attending high-performing schools had lower rates of risky health behaviors, particularly substance use. Notably, another cross-sectional study^26^ suggested that students who perceived greater support from teachers were less likely to engage in NSSI. A supportive school environment is characterized by strong teacher-student relationships and a sense of belonging to the school. Theoretical frameworks suggest that in such environments, students are more likely to be engaged academically and feel a connection to the school community. This engagement is thought to foster the development of emotional and social skills and promote positive relationships, which, in turn, enhance mental health, buffer against adverse outcomes associated with ACEs, and reduce the risk of NSSI and suicidality.^24,55^

### Strength and Limitations

The primary strength of our study is the use of a nationally representative sample of Chinese adolescents, which provides sufficient statistical power and broad generalizability, enabling a robust investigation of associations between ACEs and outcomes (ie, NSSI, SI, and SA), considering school environments. However, several limitations should be noted. First, owing to the cross-sectional nature of our study design, we can draw no causal conclusions concerning ACEs and NSSI and suicidality. Second, the reliance on self-reported data for ACEs, supportive school environments, NSSI, and suicidality may introduce the possibility of recall bias. Third, the study sample primarily consisted of school-attending adolescents, which may limit the generalizability of findings to all Chinese adolescents. Fourth, although we used a directed acyclic graph to identify a plausible set of confounders, our study may still not have accounted for all confounders, such as parenting style.

## Conclusions

In this cross-sectional study of a nationally representative sample of school-based adolescents, we identified independent associations of cumulative and specific subtypes of ACEs with NSSI and suicidality among adolescents. A dose-response association was observed, with cumulative ACEs associated with increased risks of NSSI and suicidality. Notably, threat-related ACEs, particularly emotional abuse, exhibited associations with the highest increases in risk of these outcomes. Additionally, a supportive school environment was found to mitigate associations of ACEs with NSSI and suicidality. These findings underscore the association of ACEs and school environments with these outcomes and suggest that interventions targeting ACEs, along with school-level interventions, may be effective in reducing risks of NSSI and suicidality among adolescents.

## Notes

Supplement 1. **eMethods**. Supplemental methods **eFigure 1.** Flowchart of participant selection **eFigure 2.** Directed acyclic graph depicting the appropriate covariate set **eTable 1.** Prevalence and 95% CIs of NSSI, suicidal ideation, and suicide attempts among study participants **eTable 2.** Associations of cumulative ACEs with NSSI, suicidal ideation, and suicide attempt in males **eTable 3.** Associations of cumulative ACEs with NSSI, suicidal ideation, and suicide attempt in females **eTable 4. **Associations of each ACE subtype with NSSI, suicidal ideation, and suicide attempt in males **eTable 5.** Associations of each ACE subtype with NSSI, suicidal ideation, and suicide attempt in females **eTable 6.** Associations of supportive school environments with NSSI, suicidal ideation, and suicide attempt in males **eTable 7.** Associations of supportive school environments with NSSI, suicidal ideation, and suicide attempt in females **eTable 8.** Interactions between ACEs and supportive school environments in males **eTable 9.** Interactions between ACEs and supportive school environments in females **eTable 10.** Associations between cumulative ACEs and NSSI (defined as reporting 5 or more times) **eTable 11.** Associations between each ACE subtype and NSSI (defined as reporting 5 or more times) **eTable 12. **Interactions of ACEs and supportive school environments with NSSI (defined as reporting 5 or more times) **eTable 13.** Associations of cumulative ACEs with NSSI, suicidal ideation, and suicide attempt, further adjusted for depressive symptoms **eTable 14.** Associations of each ACE subtype with NSSI, suicidal ideation, and suicide attempt, further adjusted for depressive symptoms **eTable 15.** Associations of supportive school environments with NSSI, suicidal ideation, and suicide attempt, further adjusted for depressive symptoms **eTable 16. **Interactions between ACEs and supportive school environments, further adjusted for depressive symptoms
eReferences.

Supplement 2.
Data Sharing Statement
