# Supplementary material for: Adverse Childhood Experiences and Nonsuicidal Self-Injury and Suicidality in Chinese Adolescents
Source: JAMA Netw Open. 2024 Dec 30;7(12):e2452816. doi: 10.1001/jamanetworkopen.2024.52816 (PMC11686413; doi:10.1001/jamanetworkopen.2024.52816)
Supplement: Supplement 1. — eMethods. Supplemental methods eFigure 1. Flowchart of participant selection eFigure 2. Directed acyclic graph depicting the appropriate covariate set eTable 1. Prevalence and 95% CIs of NSSI, suicidal ideation, and suicide attempts among study participants eTable 2. Associations of cumulative ACEs with NSSI, suicidal ideation, and suicide attempt in males eTable 3. Associations of cumulative ACEs with NSSI, suicidal ideation, and suicide attempt in females eTable 4. Associations of each ACE subtype with NSSI, suicidal ideation, and suicide attempt in males eTable 5. Associations of each ACE subtype with NSSI, suicidal ideation, and suicide attempt in females eTable 6. Associations of supportive school environments with NSSI, suicidal ideation, and suicide attempt in males eTable 7. Associations of supportive school environments with NSSI, suicidal ideation, and suicide attempt in females eTable 8. Interactions between ACEs and supportive school environments in males eTable 9. Interactions between ACEs and supportive school environments in females eTable 10. Associations between cumulative ACEs and NSSI (defined as reporting 5 or more times) eTable 11. Associations between each ACE subtype and NSSI (defined as reporting 5 or more times) eTable 12. Interactions of ACEs and supportive school environments with NSSI (defined as reporting 5 or more times) eTable 13. Associations of cumulative ACEs with NSSI, suicidal ideation, and suicide attempt, further adjusted for depressive symptoms eTable 14. Associations of each ACE subtype with NSSI, suicidal ideation, and suicide attempt, further adjusted for depressive symptoms eTable 15. Associations of supportive school environments with NSSI, suicidal ideation, and suicide attempt, further adjusted for depressive symptoms eTable 16. Interactions between ACEs and supportive school environments, further adjusted for depressive symptoms eReferences. [file jamanetwopen-e2452816-s001.pdf]

Supplemental Online Content

He Y, Jiang W, Wang W, Liu Q, Peng S, Guo L. Adverse childhood experiences and nonsuicidal self-injury and suicidality in Chinese adolescents. *JAMA Netw Open*. 2024;7(12):e2452816. doi:10.1001/jamanetworkopen.2024.52816

eMethods. Supplemental methods

eFigure 1. Flowchart of participant selection

eFigure 2. Directed acyclic graph depicting the appropriate covariate set

eTable 1. Prevalence and 95% CIs of NSSI, suicidal ideation, and suicide attempts among study participants

eTable 2. Associations of cumulative ACEs with NSSI, suicidal ideation, and suicide attempt in males

eTable 3. Associations of cumulative ACEs with NSSI, suicidal ideation, and suicide attempt in females

eTable 4. Associations of each ACE subtype with NSSI, suicidal ideation, and suicide attempt in males

eTable 5. Associations of each ACE subtype with NSSI, suicidal ideation, and suicide attempt in females

eTable 6. Associations of supportive school environments with NSSI, suicidal ideation, and suicide attempt in males

eTable 7. Associations of supportive school environments with NSSI, suicidal ideation, and suicide attempt in females

© 2024 He Y et al. *JAMA Network Open.*

eTable 8. Interactions between ACEs and supportive school environments in males

eTable 9. Interactions between ACEs and supportive school environments in females

eTable 10. Associations between cumulative ACEs and NSSI (defined as reporting 5 or more times)

eTable 11. Associations between each ACE subtype and NSSI (defined as reporting 5 or more times)

eTable 12. Interactions of ACEs and supportive school environments with NSSI (defined as reporting 5 or more times)

eTable 13. Associations of cumulative ACEs with NSSI, suicidal ideation, and suicide attempt, further adjusted for depressive symptoms

eTable 14. Associations of each ACE subtype with NSSI, suicidal ideation, and suicide attempt, further adjusted for depressive symptoms

eTable 15. Associations of supportive school environments with NSSI, suicidal ideation, and suicide attempt, further adjusted for depressive symptoms

eTable 16. Interactions between ACEs and supportive school environments, further adjusted for depressive symptoms

eReferences.

This supplemental material has been provided by the authors to give readers additional information about their work.

© 2024 He Y et al. *JAMA Network Open.*

eMethods. Supplemental Methods.

Study design and population

The present study used data from the 2021 School-Based Chinese Adolescents Health Survey (SCAHS), an ongoing survey of health-related behaviors among Chinese adolescents in grades 7 through 12 [1]. The 2021 SCAHS used a multi-stage, stratified cluster random sampling method, and the procedures for data collection were as follows. In stage 1, we divided Chinese provinces into three economic strata (high-economic level, middle-economic level, low-economic level) according to per capita gross domestic product (GDP) level. Based on the proportion of each stratum and cooperation, eight provinces were selected to participate in our study: Guangdong, Henan, and Shandong (high-level); Chongqing and Yunnan (middle-level); Guizhou, Liaoning, and Heilongjiang (low-level). With the assistance of local education bureaus, seven cities in Guangdong and three cities in other provinces were randomly selected. In stage 2, six junior high schools, four senior high schools, and two vocational high schools were randomly selected based on the overall proportion of the three types of schools in the chosen cities. In stage 3, two classes were randomly selected from each grade within the chosen schools. All available students in the selected classes except those who suffered from severe mental or physical disorders, as identified by the head teacher and/or health care physicians, were invited to participate in the study. We ensured that a detailed written informed consent was provided to both students and their legal guardians. The survey was conducted by trained investigators available to address any queries or confusion the participants might have had regarding the structured

© 2024 He Y et al. *JAMA Network Open.*

questionnaire. Participants completed the survey within a single session at school after being informed of the study's objectives and procedures. To minimize potential external influences on students' responses, and protect students' privacy, all participants were expected to complete the anonymous questionnaire within 45 minutes independently without the presence of teachers. Furthermore, the completeness and quality of the questionnaires were reviewed by the investigator at the end of the survey to ensure the accuracy and reliability of the data collected.

Adverse Childhood Experiences (ACEs)

Based on the Centers for Disease Control and Prevention (CDC)-Kaiser Permanente ACE study [2, 3] and the ACE-IQ [4], we collected 15 conventional and expanded forms of ACEs by questionnaire using either scales validated in Chinese adolescents or specific questions widely used in previous studies [3]. Moreover, these ACEs can be categorized into threat-related and deprivation-related ACEs. Threat-related ACEs were considered as events that were physically or mentally threatening to the respondents, including physical abuse, emotional abuse, sexual abuse, household substance abuse, witness of community violence, witness of domestic violence, sex discrimination, and bullying. Deprivation-related ACEs reflect some of the long-lasting unmet needs, both material and psychological, that include emotional neglect, physical neglect, household mental illness, incarcerated household member, parental separation or divorce, family financial problems, and parent death[5, 6]. The detailed questionnaire items and definitions of each ACE indicator are as follows.

© 2024 He Y et al. *JAMA Network Open.*

Neglect (emotional neglect and physical neglect) and abuse (emotional abuse, physical abuse, and sex abuse) were assessed by using the simplified version of the Childhood Trauma Questionnaire (CTQ-SF) [7], a 28-item self-report questionnaire widely used in Chinese adolescents with good reliability and validity[8]. It contained five subscales to assess sex abuse, physical abuse, emotional abuse, physical neglect, and emotional neglect. Each item of CTQ-SF related to a 5-point Likert scale ranging from "never" to "very often". The sum of these items provides an overall CTQ-SF score, a higher score means a greater impact of the ACEs. We calculated the total score of each subscale of CTQ-SF, and according to the cut-off scores of each subscale (emotional abuse score ≥ 13, physical abuse score ≥ 10, sexual abuse score ≥ 8, emotional neglect score ≥ 15, and physical neglect score ≥ 10)[9], participants were classified into whether they experienced corresponding subtype of ACEs. The CTQ-SF exhibited high internal consistency in the present study, with a Cronbach's α of 0.83.

While the other 10 subtypes of ACEs were measured with a single item adapted from the original ACE study [33]. Participants were asked whether they had the following experiences during their lives: bullying, parental divorce, household criminality, household mental illness, household substance use, family financial problems, parent death, witness of community violence, witness of domestic violence, and sex discrimination. The responses of these items were coded as "No" =0, and "Yes"= 1.

Non-suicidal Self-injury (NSSI)

NSSI was assessed using the Chinese version of the Function Assessment of Self-

© 2024 He Y et al. *JAMA Network Open.*

Mutilation [10], which demonstrated a Cronbach's α of 0.78 in this study.

Depressive symptoms

Depressive symptoms were measured using the 20-item version of the Center for Epidemiologic Studies Depression Scale (CESD-20), which has been widely used in Chinese adolescents with good reliability and validity [1, 11]. Each item is scored from 0 to 3, with a total score ranging from 0 to 60. Higher scores indicate more severe depressive symptomatology [1, 11]. The Cronbach's α in the present study was 0.87.

Statistical analysis

In cases where the outcome is common (e.g., prevalence exceeds 10%), using ORs may substantially overestimate the association strength [12-14]. In our study, the relatively high prevalence of NSSI and suicidal ideation made PRs a preferable choice, as ORs would yield biased estimates in this context. Additionally, while ORs approximate PRs when prevalence is below 10%, PRs provide a more interpretable measure by directly indicating the relative increase in prevalence [14]. This approach is well-suited for our cross-sectional study [15]. Although the prevalence of suicide attempts in our sample is lower (approximately 5%), we opted to calculate PRs consistently across outcomes for comparability. Therefore, weighted Poisson regression models that accounted for the stratified cluster survey design were performed to test the associations of ACEs and supportive school environments with NSSI, SI, and SA, and prevalence ratios (PRs) with 95% CIs were estimated.

© 2024 He Y et al. *JAMA Network Open.*

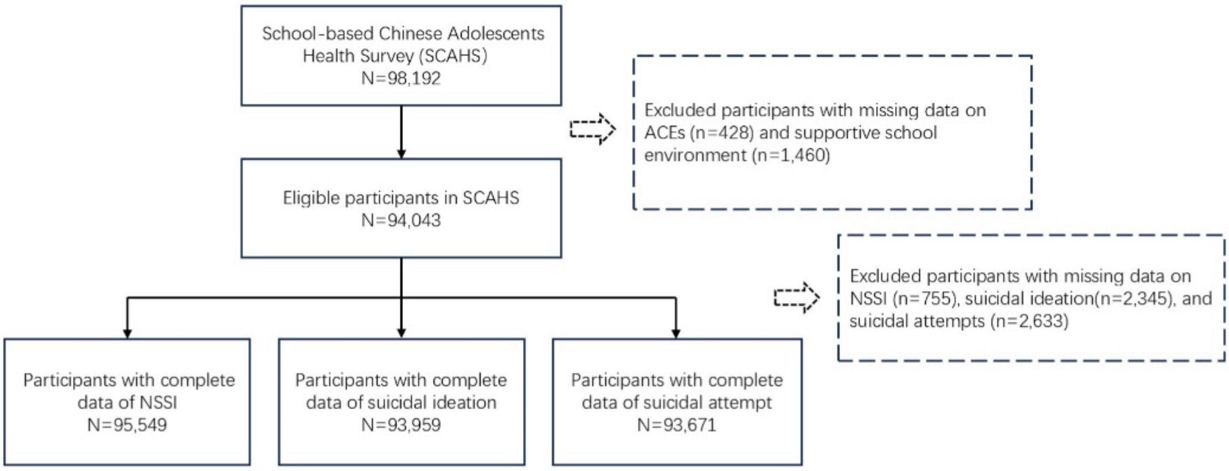

eFigure 1. Flowchart of participant selection.

© 2024 He Y et al. *JAMA Network Open.*

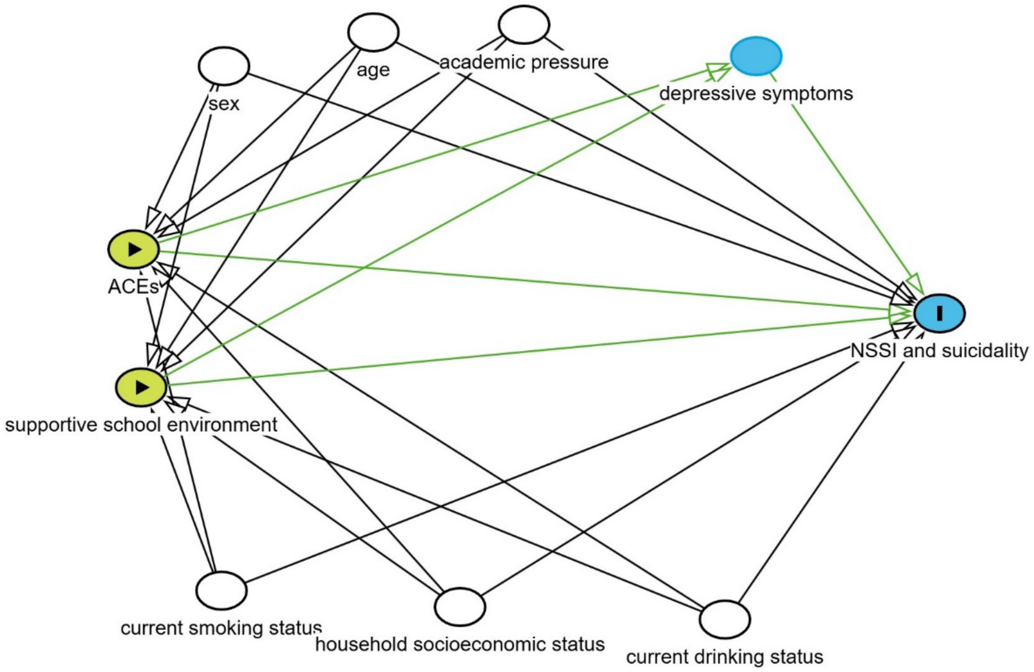

eFigure 2. Directed acyclic graph (DAG) depicting the appropriate covariate set.
Note: The final set of covariates for the main analysis included age, sex, current drinking status, current smoking status, household socioeconomic status, and academic pressure.

© 2024 He Y et al. *JAMA Network Open.*

eTable 1. Prevalence and 95% confidence interval of NSSI, suicidal ideation, and suicide attempts among study participants.

Variable	NSSI, % (95%CI)	Suicidal ideation, % (95%CI)	Suicide attempt, % (95%CI)
Overall	15.0 (14.8-15.3)	18.7 (18.4-19.0)	5.6 (5.5-5.8)
Sex			
Male	11.2 (10.9-11.6)	13.3 (12.9-13.6)	3.5 (3.3-3.7)
Female	19.2 (18.8-19.6)	24.7 (24.2-25.2)	8.0 (7.7-8.3)
SES			
Above average	13.0 (12.5-13.4)	15.9 (15.4-16.5)	5.1 (4.8-5.4)
Average	14.9 (14.6-15.2)	18.7 (18.3-19.1)	5.4 (5.2-5.6)
Below average	19.9 (19.1-20.6)	25.0 (24.1-25.8)	8.1 (7.5-8.6)
Current smoking status			
No	14.6 (14.3-14.8)	18.1 (17.8-17.4)	5.2 (5.0-5.4)
Yes	23.5 (22.2-24.9)	30.4 (29.0-31.9)	13.9 (12.8-15.0)
Current drinking status			
No	14.6 (14.3-14.8)	18.1 (17.9-18.4)	5.2 (5.1-5.4)
Yes	24.3 (22.9-25.7)	31.1 (29.6-32.6)	14.2 (13.1-15.4)
Academic pressure			
None	8.3 (7.8-8.7)	10.4 (9.9-10.9)	3.2 (2.9-3.5)
Moderate	12.5 (12.1-12.8)	15.1 (14.7-15.4)	4.3 (4.0-4.5)
Severe	22.7 (22.2-23.3)	29.0 (28.4-29.6)	0.9 (0.8-0.9)
Any ACEs-No.			
0	8.1 (7.8-8.3)	9.2 (8.9-9.5)	1.8 (1.6-1.9)
≥1	23.2 (22.7-23.7)	29.9 (29.3-30.4)	10.1 (9.8-10.5)
No. of ACEs			
0	8.1 (7.8-8.3)	9.2 (8.9-9.5)	1.8 (1.6-1.9)
1	15.8 (15.2-16.4)	20.2 (19.3-20.5)	5.0 (4.6-5.3)
2	21.4 (20.6-22.3)	28.0 (27.1-29.0)	8.4 (7.8-9.0)
3	33.4 (31.9-34.8)	42.7 (41.2-44.3)	15.3 (14.1-16.4)
≥4	48.1 (46.7-49.7)	62.2 (60.8-63.8)	30.5 (29.0-32.0)
No. of threat-related ACEs			
0	9.1 (8.8-9.3)	11.1 (10.8-11.4)	2.5 (2.4-2.6)
1	24.7 (23.9-25.5)	31.2 (30.4-32.1)	9.1 (8.6-9.6)
≥2	44.6 (43.5-45.8)	55.6 (54.4-56.8)	24.0 (23.0-25.0)
No. of deprivation-related ACEs			
0	11.6 (11.3-11.9)	13.6 (13.2-13.9)	3.2 (3.0-3.4)
1	18.8 (18.2-19.5)	24.6 (23.8-25.2)	7.4 (7.0-7.8)
≥2	26.7 (25.8-27.6)	36.4 (35.4-37.4)	15.5 (14.7-16.2)

Abbreviations: NSSI, non-suicidal self-injury; ACE, adverse childhood experience.

© 2024 He Y et al. *JAMA Network Open.*

eTable 2. Associations between cumulative ACEs with NSSI, suicidal ideation, and suicide attempt in males.

Variable	PR (95% CI)					
	NSSI		Suicidal ideation		Suicide attempt	
	Model 1[a]	Model 2[b]	Model 1[a]	Model 2[b]	Model 1[a]	Model 2[b]
The cumulative of ACEs (1-unit increase)	1.33 (1.31-1.35)	1.05 (1.04-1.05)	1.36 (1.35-1.38)	1.06 (1.06-1.06)	1.51 (1.48-1.55)	1.03 (1.03-1.03)
Any ACEs, No.						
0	1 (Reference)	1 (Reference)	1 (Reference)	1 (Reference)	1 (Reference)	1 (Reference)
≥1	2.55 (2.38-2.72)	1.08 (1.08-1.09)	3.13 (2.93-3.34)	1.12 (1.11-1.12)	5.11 (4.36-5.98)	1.04 (1.04-1.05)
No. of ACEs						
0	1 (Reference)	1 (Reference)	1 (Reference)	1 (Reference)	1 (Reference)	1 (Reference)
1	1.88 (1.73-2.04)	1.05 (1.04-1.05)	2.19 (2.03-2.37)	1.06 (1.05-1.07)	2.80 (2.31-3.39)	1.02 (1.01-1.02)
2	2.37 (2.17-2.59)	1.07 (1.06-1.08)	2.99 (2.76-3.25)	1.11 (1.1-1.12)	4.37 (3.60-5.31)	1.03 (1.03-1.04)
3	3.69 (3.35-4.07)	1.16 (1.14-1.17)	4.56 (4.18-4.98)	1.21 (1.19-1.23)	7.71 (6.28-9.45)	1.07 (1.06-1.09)
≥4	5.20 (4.76-5.67)	1.26 (1.24-1.28)	6.63 (6.12-7.18)	1.36 (1.33-1.38)	16.28 (13.58-19.51)	1.19 (1.17-1.2)
The cumulative of threat-related ACEs	1.59 (1.56-1.62)	1.09 (1.08-1.09)	1.59 (1.56-1.62)	1.10 (1.10-1.11)	1.83 (1.77-1.88)	1.05 (1.05-1.05)
No. of threat-related ACEs						
0	1 (Reference)	1 (Reference)	1 (Reference)	1 (Reference)	1 (Reference)	1 (Reference)
1	2.56 (2.38-2.75)	1.09 (1.09-1.10)	2.75 (2.58-2.93)	1.12 (1.11-1.13)	3.33 (2.87-3.87)	1.03 (1.03-1.04)
≥2	4.71 (4.39-5.05)	1.25 (1.23-1.26)	5.00 (4.69-5.32)	1.30 (1.28-1.32)	9.08 (7.90-10.44)	1.13 (1.12-1.15)
The cumulative of deprivation-related ACEs	1.29 (1.25-1.32)	1.03 (1.02-1.03)	1.42 (1.39-1.46)	1.05 (1.05-1.05)	1.74 (1.66-1.82)	1.03 (1.02-1.03)
No. of deprivation-related ACEs						
0	1 (Reference)	1 (Reference)	1 (Reference)	1 (Reference)	1 (Reference)	1 (Reference)
1	1.39 (1.29-1.49)	1.03 (1.02-1.04)	1.65 (1.54-1.75)	1.05 (1.04-1.06)	2.07 (1.79-2.39)	1.02 (1.01-1.02)
≥2	1.76 (1.63-1.89)	1.06 (1.05-1.07)	2.27 (2.13-2.42)	1.11 (1.10-1.12)	3.90 (3.41-4.46)	1.06 (1.05-1.07)

Abbreviations: ACE, adverse childhood experience; NSSI, non-suicidal self-injury; PR, prevalence ratio.

[a] Model 1 was adjusted for age, sex, current drinking status, current smoking status, household socioeconomic status, and academic pressure.

[b] Model 2 was adjusted for the variables in Model 1, plus supportive school environments.

© 2024 He Y et al. *JAMA Network Open.*

eTable 3. Associations between cumulative ACEs with NSSI, suicidal ideation, and suicide attempt in females.

Variable	PR (95% CI)					
	NSSI		Suicidal ideation		Suicide attempt	
	Model 1[a]	Model 2[b]	Model 1[a]	Model 2[b]	Model 1[a]	Model 2[b]
The cumulative of ACEs (1-unit increase)	1.29 (1.28-1.31)	1.06 (1.06-1.06)	1.3 (1.29-1.31)	1.08 (1.07-1.08)	1.44 (1.42-1.46)	1.05 (1.05-1.05)
Any ACEs, No.						
0	1 (Reference)	1 (Reference)	1 (Reference)	1 (Reference)	1 (Reference)	1 (Reference)
≥1	2.55 (2.43-2.68)	1.14 (1.13-1.15)	2.74 (2.62-2.86)	1.19 (1.18-1.20)	4.68 (4.24-5.17)	1.09 (1.08-1.10)
No. of ACEs						
0	1 (Reference)	1 (Reference)	1 (Reference)	1 (Reference)	1 (Reference)	1 (Reference)
1	1.84 (1.73-1.96)	1.07 (1.06-1.08)	1.98 (1.88-2.09)	1.10 (1.09-1.11)	2.58 (2.29-2.92)	1.04 (1.03-1.04)
2	2.53 (2.37-2.70)	1.14 (1.12-1.15)	2.75 (2.61-2.91)	1.19 (1.18-1.20)	4.35 (3.85-4.91)	1.08 (1.07-1.09)
3	3.57 (3.34-3.83)	1.24 (1.22-1.26)	3.80 (3.59-4.02)	1.31 (1.29-1.33)	7.30 (6.45-8.26)	1.16 (1.15-1.18)
≥4	4.57 (4.30-4.85)	1.35 (1.33-1.37)	4.86 (4.62-5.10)	1.45 (1.43-1.47)	11.82 (10.57-13.22)	1.31 (1.29-1.33)
The cumulative of threat-related ACEs	1.46 (1.44-1.48)	1.10 (1.09-1.10)	1.44 (1.43-1.46)	1.11 (1.11-1.12)	1.65 (1.62-1.68)	1.08 (1.07-1.08)
No. of threat-related ACEs						
0	1 (Reference)	1 (Reference)	1 (Reference)	1 (Reference)	1 (Reference)	1 (Reference)
1	2.42 (2.30-2.55)	1.15 (1.14-1.16)	2.42 (2.31-2.52)	1.18 (1.17-1.20)	3.22 (2.94-3.54)	1.07 (1.07-1.08)
≥2	3.69 (3.51-3.87)	1.30 (1.28-1.31)	3.61 (3.47-3.76)	1.36 (1.35-1.37)	6.80 (6.22-7.42)	1.22 (1.21-1.24)
The cumulative of deprivation-related ACEs	1.37 (1.35-1.40)	1.06 (1.05-1.06)	1.42 (1.4-1.45)	1.08 (1.08-1.09)	1.70 (1.65-1.75)	1.06 (1.05-1.06)
No. of deprivation-related ACEs						
0	1 (Reference)	1 (Reference)	1 (Reference)	1 (Reference)	1 (Reference)	1 (Reference)
1	1.57 (1.49-1.65)	1.06 (1.06-1.07)	1.67 (1.60-1.75)	1.09 (1.08-1.10)	2.11 (1.93-2.32)	1.04 (1.04-1.05)
≥2	2.16 (2.05-2.28)	1.14 (1.13-1.15)	2.39 (2.29-2.49)	1.20 (1.19-1.22)	4.08 (3.74-4.45)	1.14 (1.13-1.15)

Abbreviations: ACE, adverse childhood experience; NSSI, non-suicidal self-injury; PR, prevalence ratio.

[a] Model 1 was adjusted for age, sex, current drinking status, current smoking status, household socioeconomic status, and academic pressure.

[b] Model 2 was adjusted for the variables in Model 1, plus supportive school environments.

© 2024 He Y et al. *JAMA Network Open.*

eTable 4. Associations between each ACE subtype with NSSI, suicidal ideation, and suicide attempt in males.

ACE subtype	PR (95% CI)					
	NSSI		Suicidal ideation		Suicide attempt	
	Model 1[a]	Model 2[b]	Model 1[a]	Model 2[b]	Model 1[a]	Model 2[b]
Threat-related ACEs						
Physical abuse						
No	1 (Reference)	1 (Reference)	1 (Reference)	1 (Reference)	1 (Reference)	1 (Reference)
Yes	3.05 (2.80-3.33)	1.22 (1.20-1.25)	3.22 (3.00-3.46)	1.29 (1.26-1.32)	5.20 (4.49-6.01)	1.16 (1.14-1.19)
Sex abuse						
No	1 (Reference)	1 (Reference)	1 (Reference)	1 (Reference)	1 (Reference)	1 (Reference)
Yes	2.78 (2.54-3.05)	1.19 (1.17-1.22)	2.50 (2.30-2.72)	1.20 (1.17-1.22)	3.59 (3.04-4.23)	1.11 (1.09-1.13)
Emotional abuse						
No	1 (Reference)	1 (Reference)	1 (Reference)	1 (Reference)	1 (Reference)	1 (Reference)
Yes	3.12 (2.89-3.36)	1.22 (1.20-1.24)	3.37 (3.16-3.58)	1.30 (1.27-1.32)	5.63 (4.93-6.42)	1.16 (1.14-1.18)
Household substance abuse						
No	1 (Reference)	1 (Reference)	1 (Reference)	1 (Reference)	1 (Reference)	1 (Reference)
Yes	2.46 (2.09-2.90)	1.16 (1.12-1.21)	2.88 (2.54-3.26)	1.25 (1.21-1.30)	4.68 (3.67-5.97)	1.16 (1.11-1.20)
Witness of community violence						
No	1 (Reference)	1 (Reference)	1 (Reference)	1 (Reference)	1 (Reference)	1 (Reference)
Yes	2.58 (2.39-2.78)	1.15 (1.13-1.17)	2.34 (2.19-2.51)	1.15 (1.13-1.17)	3.16 (2.74-3.64)	1.07 (1.06-1.08)
Sex discrimination						
No	1 (Reference)	1 (Reference)	1 (Reference)	1 (Reference)	1 (Reference)	1 (Reference)
Yes	2.68 (2.40-3.00)	1.19 (1.15-1.22)	2.44 (2.21-2.70)	1.19 (1.16-1.22)	3.41 (2.78-4.19)	1.10 (1.07-1.12)
Bullying						
No	1 (Reference)	1 (Reference)	1 (Reference)	1 (Reference)	1 (Reference)	1 (Reference)
Yes	2.71 (2.55-2.88)	1.14 (1.13-1.15)	2.55 (2.42-2.70)	1.15 (1.14-1.16)	3.46 (3.08-3.90)	1.06 (1.05-1.07)
Deprivation-related ACEs						
Physical neglect						
No	1 (Reference)	1 (Reference)	1 (Reference)	1 (Reference)	1 (Reference)	1 (Reference)
Yes	1.29 (1.21-1.38)	1.02 (1.01-1.03)	1.60 (1.51-1.69)	1.06 (1.05-1.06)	2.29 (2.04-2.56)	1.03 (1.03-1.04)

© 2024 He Y et al. *JAMA Network Open.*

eTable 4. Associations between each ACE subtype with NSSI, suicidal ideation, and suicide attempt in males (continued).

Emotional neglect						
No	1 (Reference)	1 (Reference)	1 (Reference)	1 (Reference)	1 (Reference)	1 (Reference)
Yes	1.63 (1.52-1.75)	1.05 (1.04-1.06)	1.94 (1.83-2.06)	1.1 (1.08-1.11)	2.93 (2.60-3.31)	1.05 (1.05-1.06)
Parental divorce						
No	1 (Reference)	1 (Reference)	1 (Reference)	1 (Reference)	1 (Reference)	1 (Reference)
Yes	1.33 (1.23-1.45)	1.03 (1.02-1.04)	1.60 (1.49-1.71)	1.07 (1.06-1.08)	1.87 (1.62-2.15)	1.03 (1.02-1.04)
Household criminality						
No	1 (Reference)	1 (Reference)	1 (Reference)	1 (Reference)	1 (Reference)	1 (Reference)
Yes	1.52 (1.26-1.83)	1.05 (1.02-1.08)	1.94 (1.68-2.25)	1.11 (1.08-1.15)	2.28 (1.70-3.05)	1.05 (1.02-1.07)
Household domestic violence						
No	1 (Reference)	1 (Reference)	1 (Reference)	1 (Reference)	1 (Reference)	1 (Reference)
Yes	2.27 (2.11-2.43)	1.12 (1.10-1.13)	2.65 (2.50-2.81)	1.18 (1.16-1.19)	3.38 (2.97-3.85)	1.07 (1.06-1.08)
Household mental illness						
No	1 (Reference)	1 (Reference)	1 (Reference)	1 (Reference)	1 (Reference)	1 (Reference)
Yes	2.43 (2.06-2.86)	1.16 (1.11-1.20)	2.62 (2.29-2.98)	1.21 (1.17-1.26)	4.16 (3.24-5.34)	1.13 (1.09-1.17)
Family financial problems						
No	1 (Reference)	1 (Reference)	1 (Reference)	1 (Reference)	1 (Reference)	1 (Reference)
Yes	1.69 (1.53-1.87)	1.07 (1.05-1.09)	1.67 (1.53-1.82)	1.08 (1.07-1.10)	1.69 (1.26-2.27)	1.04 (1.03-1.05)
Parental death						
No	1 (Reference)	1 (Reference)	1 (Reference)	1 (Reference)	1 (Reference)	1 (Reference)
Yes	1.19 (1.00-1.42)	1.02 (1.01-1.04)	1.23 (1.05-1.44)	1.03 (1.01-1.06)	1.69 (1.26-2.27)	1.03 (1.01-1.05)

Abbreviations: ACE, adverse childhood experience; NSSI, non-suicidal self-injury; PR, prevalence ratio.

[a] Model 1 was adjusted for age, sex, current drinking status, current smoking status, household socioeconomic status, and academic pressure.

[b] Model 2 was adjusted for the variables in Model 1, plus supportive school environments.

© 2024 He Y et al. *JAMA Network Open.*

eTable 5. Associations between each ACE subtype with NSSI, suicidal ideation, and suicide attempt in females.

ACE subtype	PR (95% CI)					
	NSSI		Suicidal ideation		Suicide attempt	
	Model 1[a]	Model 2[b]	Model 1[a]	Model 2[b]	Model 1[a]	Model 2[b]
Threat-related ACEs						
Physical abuse						
No	1 (Reference)	1 (Reference)	1 (Reference)	1 (Reference)	1 (Reference)	1 (Reference)
Yes	2.63 (2.47-2.80)	1.29 (1.26-1.31)	2.48 (2.36-2.61)	1.31 (1.29-1.33)	3.93 (3.54-4.36)	1.26 (1.23-1.29)
Sex abuse						
No	1 (Reference)	1 (Reference)	1 (Reference)	1 (Reference)	1 (Reference)	1 (Reference)
Yes	2.41 (2.23-2.60)	1.24 (1.21-1.27)	2.13 (2.00-2.28)	1.23 (1.20-1.26)	3.43 (3.04-3.87)	1.21 (1.18-1.24)
Emotional abuse						
No	1 (Reference)	1 (Reference)	1 (Reference)	1 (Reference)	1 (Reference)	1 (Reference)
Yes	2.71 (2.59-2.84)	1.27 (1.25-1.29)	2.82 (2.72-2.93)	1.34 (1.33-1.36)	4.75 (4.40-5.12)	1.25 (1.23-1.27)
Household substance abuse						
No	1 (Reference)	1 (Reference)	1 (Reference)	1 (Reference)	1 (Reference)	1 (Reference)
Yes	2.26 (2.04-2.51)	1.21 (1.17-1.25)	2.07 (1.90-2.25)	1.21 (1.18-1.25)	2.46 (2.05-2.96)	1.12 (1.08-1.16)
Witness of community violence						
No	1 (Reference)	1 (Reference)	1 (Reference)	1 (Reference)	1 (Reference)	1 (Reference)
Yes	1.78 (1.47-2.16)	1.14 (1.12-1.16)	1.76 (1.68-1.85)	1.14 (1.12-1.16)	2.21 (2.00-2.43)	1.08 (1.07-1.10)
Sex discrimination						
No	1 (Reference)	1 (Reference)	1 (Reference)	1 (Reference)	1 (Reference)	1 (Reference)
Yes	2.09 (1.97-2.21)	1.17 (1.15-1.19)	2.04 (1.95-2.13)	1.20 (1.18-1.22)	2.52 (2.29-2.77)	1.11 (1.10-1.13)
Bullying						
No	1 (Reference)	1 (Reference)	1 (Reference)	1 (Reference)	1 (Reference)	1 (Reference)
Yes	2.33 (2.23-2.44)	1.19 (1.18-1.21)	2.23 (2.15-2.32)	1.22 (1.20-1.23)	3.14 (2.91-3.40)	1.14 (1.13-1.15)
Deprivation-related ACEs						
Physical neglect						
No	1 (Reference)	1 (Reference)	1 (Reference)	1 (Reference)	1 (Reference)	1 (Reference)
Yes	1.59 (1.52-1.67)	1.08 (1.07-1.09)	1.69 (1.62-1.75)	1.11 (1.10-1.12)	2.48 (2.30 2.67)	1.09 (1.08-1.10)

© 2024 He Y et al. *JAMA Network Open.*

eTable 5. Associations between each ACE subtype with NSSI, suicidal ideation, and suicide attempt in females (continued).

Emotional neglect						
No	1 (Reference)	1 (Reference)	1 (Reference)	1 (Reference)	1 (Reference)	1 (Reference)
Yes	1.89 (1.80-1.98)	1.12 (1.11-1.14)	2.10 (2.03-2.19)	1.19 (1.18-1.20)	3.28 (3.05-3.54)	1.14 (1.12-1.15)
Parental divorce						
No	1 (Reference)	1 (Reference)	1 (Reference)	1 (Reference)	1 (Reference)	1 (Reference)
Yes	1.34 (1.27-1.42)	1.05 (1.04-1.06)	1.47 (1.41-1.54)	1.09 (1.08-1.10)	1.72 (1.58-1.87)	1.05 (1.04-1.06)
Household criminality						
No	1 (Reference)	1 (Reference)	1 (Reference)	1 (Reference)	1 (Reference)	1 (Reference)
Yes	1.41 (1.24-1.60)	1.06 (1.03-1.09)	1.71 (1.56-1.88)	1.14 (1.11-1.17)	1.95 (1.62-2.35)	1.08 (1.05-1.11)
Household domestic violence						
No	1 (Reference)	1 (Reference)	1 (Reference)	1 (Reference)	1 (Reference)	1 (Reference)
Yes	2.01 (1.91-2.11)	1.15 (1.14-1.17)	2.05 (1.97-2.14)	1.19 (1.18-1.21)	2.67 (2.46-2.90)	1.11 (1.10-1.13)
Household mental illness						
No	1 (Reference)	1 (Reference)	1 (Reference)	1 (Reference)	1 (Reference)	1 (Reference)
Yes	2.41 (2.20-2.64)	1.23 (1.19-1.26)	2.00 (1.84-2.17)	1.19 (1.16-1.23)	2.85 (2.43-3.34)	1.14 (1.11-1.18)
Family financial problems						
No	1 (Reference)	1 (Reference)	1 (Reference)	1 (Reference)	1 (Reference)	1 (Reference)
Yes	1.68 (1.56-1.81)	1.10 (1.09-1.12)	1.59 (1.49-1.69)	1.11 (1.09-1.13)	1.56 (1.36-1.79)	1.04 (1.02-1.06)
Parental death						
No	1 (Reference)	1 (Reference)	1 (Reference)	1 (Reference)	1 (Reference)	1 (Reference)
Yes	1.15 (1.01-1.29)	1.02 (1.01-1.04)	1.13 (1.02-1.26)	1.02 (1.01-1.05)	0.96 (0.77-1.20)	0.99 (0.98-1.01)

Abbreviations: ACE, adverse childhood experience; NSSI, non-suicidal self-injury; PR, prevalence ratio.

Model 1 was adjusted for age, sex, current drinking status, current smoking status, household socioeconomic status, and academic pressure.

Model 2 was adjusted for the variables in Model 1, plus supportive school environments.

© 2024 He Y et al. *JAMA Network Open.*

eTable 6. Associations between supportive school environments with NSSI, suicidal ideation, and suicide attempt in males.

Variable	PR (95% CI)	
	Model 1[a]	Model 2[b]
NSSI		
supportive school environment	0.67 (0.65-0.69)	0.74 (0.71-0.76)
Suicidal ideation		
supportive school environment	0.64 (0.62-0.66)	0.71 (0.69-0.73)
Suicide attempt		
supportive school environment	0.57 (0.54-0.60)	0.65 (0.61-0.69)

Abbreviations: NSSI, non-suicidal self-injury; PR, prevalence ratio.

[a]Model 1 was unadjusted.

[b]Model 2 was adjusted for age, sex, current drinking status, current smoking status, household socioeconomic status, and academic pressure.

© 2024 He Y et al. *JAMA Network Open.*

eTable 7. Associations between supportive school environments with NSSI, suicidal ideation, and suicide attempt in females.

Variable	PR (95% CI)	
	Model 1[a]	Model 2[b]
NSSI		
supportive school environment	0.70 (0.68-0.71)	0.75 (0.74-0.77)
Suicidal ideation		
supportive school environment	0.71 (0.69-0.72)	0.76 (0.75-0.77)
Suicide attempt		
supportive school environment	0.61 (0.59-0.63)	0.66 (0.64-0.69)

Abbreviations: NSSI, non-suicidal self-injury; PR, prevalence ratio.

[a]Model 1 was unadjusted.

[b]Model 2 was adjusted for age, sex, current drinking status, current smoking status, household socioeconomic status, and academic pressure.

© 2024 He Y et al. *JAMA Network Open.*

eTable 8. The interactions between ACEs and supportive school environments in males.

Variable	PR (95% CI)	
	Model 1[b]	Model 2[c]
NSSI		
ACEs	2.08 (1.81-2.38)	1.95 (1.70-2.24)
supportive school environment	0.69 (0.65-0.73)	0.73 (0.69-0.78)
ACEs* supportive school environment	1.12 (1.05-1.20)	1.11 (1.04-1.19)
Interaction ratio[a]	0.78 (0.69-0.89)	0.77 (0.68-0.89)
Suicidal ideation		
ACEs	2.43 (2.13-2.77)	2.22 (1.95-2.53)
supportive school environment	0.65 (0.61-0.69)	0.70 (0.66-0.74)
ACEs* supportive school environment	1.16 (1.09-1.25)	1.16 (1.09-1.24)
Interaction ratio[a]	0.73 (0.65-0.84)	0.75 (0.66-0.84)
Suicide attempt		
ACEs	4.95 (3.56-6.87)	4.44 (3.19-6.19)
supportive school environment	0.66 (0.57-0.77)	0.72 (0.61-0.84)
ACEs* supportive school environment	1.02 (0.86-1.21)	1.02 (0.86-1.21)
Interaction ratio[a]	0.31 (0.23-0.42)	0.32 (0.23-0.44)

Abbreviations: ACEs, adverse childhood experience; NSSI, non-suicidal self-injury, PR, prevalence ratio.

[a] calculated as $PR_{\text{ACE* supportive school environment}} / (PR_{\text{ACEs}}\ PR_{\text{supportive school environment}})$

[b]Model 1 was unadjusted.

[c]Model 2 was adjusted for age, sex, current drinking status, current smoking status, household socioeconomic status, and academic pressure.

© 2024 He Y et al. *JAMA Network Open.*

eTable 9. The interactions between ACEs and supportive school environments in females.

Variable	PR (95% CI)	
	Model 1[b]	Model 2[c]
NSSI		
ACEs	2.06 (1.87-2.28)	1.83 (1.65-2.02)
supportive school environment	0.71 (0.68-0.74)	0.74 (0.71-0.77)
ACEs* supportive school environment	1.13 (1.08-1.18)	1.15 (1.09-1.20)
Interaction ratio[a]	0.77 (0.70-0.85)	0.85 (0.77-0.93)
Suicidal ideation		
ACEs	2.15 (1.97-2.35)	1.94 (1.77-2.12)
supportive school environment	0.72 (0.69-0.75)	0.74 (0.71-0.77)
ACEs* supportive school environment	1.14 (1.09-1.19)	1.16 (1.11-1.21)
Interaction ratio[a]	0.74 (0.68-0.80)	0.81 (0.74-0.88)
Suicide attempt		
ACEs	4.30 (3.54-5.22)	3.57 (2.93-4.35)
supportive school environment	0.68 (0.62-0.75)	0.70 (0.63-0.76)
ACEs* supportive school environment	1.05 (0.95-1.15)	1.09 (0.99-1.20)
Interaction ratio[a]	0.36 (0.29-0.43)	0.44 (0.36-0.54)

Abbreviations: ACEs, adverse childhood experience; NSSI, non-suicidal self-injury, PR, prevalence ratio.

[a] calculated as $PR_{\text{ACE* supportive school environment}} / (PR_{\text{ACEs}} PR_{\text{supportive school environment}})$

[b]Model 1 was unadjusted.

[c]Model 2 was adjusted for age, sex, current drinking status, current smoking status, household socioeconomic status, and academic pressure.

© 2024 He Y et al. *JAMA Network Open.*

eTable 10. Associations between cumulative ACEs and NSSI (defined as reporting 5 or more times).

Variable	PR (95% CI)	
	Model 1[a]	Model 2[b]
The cumulative of ACEs (1-unit increase)	1.06 (1.05-1.06)	1.05 (1.05-1.05)
Any ACEs		
0	1 (Reference)	1 (Reference)
≥1	1.11 (1.11-1.12)	1.10 (1.10-1.10)
No. of ACEs		
0	1 (Reference)	1 (Reference)
1	1.05 (1.05-1.06)	1.05 (1.04-1.05)
2	1.09 (1.09-1.10)	1.09 (1.08-1.09)
3	1.19 (1.18-1.20)	1.18 (1.16-1.19)
≥4	1.34 (1.32-1.35)	1.31 (1.29-1.32)
The cumulative of threat-related ACEs	1.10 (1.09-1.10)	1.09 (1.08-1.09)
No. of threat-related ACEs		
0	1 (Reference)	1 (Reference)
1	1.11 (1.10,-1.11)	1.09 (1.08-1.10)
≥2	1.29 (1.27-1.30)	1.25 (1.24-1.26)
The cumulative of deprivation-related ACEs	1.05 (1.05-1.06)	1.04 (1.04-1.04)
No. of deprivation-related ACEs		
0	1 (Reference)	1 (Reference)
1	1.05 (1.05-1.06)	1.04 (1.03-1.04)
≥2	1.12 (1.11-1.13)	1.09 (1.08,-1.10)

Abbreviations: ACE, adverse childhood experience; NSSI, non-suicidal self-injury; PR, prevalence ratio.

[a]Model 1 was adjusted for age, sex, current drinking status, current smoking status, household socioeconomic status, and academic pressure.

[b]Model 2 was adjusted for the variables in Model 1, plus supportive school environments.

© 2024 He Y et al. *JAMA Network Open.*

eTable 11. Associations between each ACE subtype and NSSI (defined as reporting 5 or more times)

ACE subtype	PR (95% CI)	
	Model 1[a]	Model 2[b]
Threaten-related ACEs		
Physical abuse		
No	1 (Reference)	1 (Reference)
Yes	1.29 (1.27-1.32)	1.26 (1.24-1.29)
Sex abuse		
No	1 (Reference)	1 (Reference)
Yes	1.24 (1.22-1.26)	1.22 (1.20-1.24)
Emotional abuse		
No	1 (Reference)	1 (Reference)
Yes	1.30(1.28-1.31)	1.26 (1.25-1.28)
Household substance abuse		
No	1 (Reference)	1 (Reference)
Yes	1.22 (1.19-1.25)	1.19 (1.16-1.23)
Witness of community violence		
No	1 (Reference)	1 (Reference)
Yes	1.15 (1.14-1.17)	1.14 (1.13-1.15)
Household domestic violence		
No	1 (Reference)	1 (Reference)
Yes	1.15 (1.14-1.16)	1.13 (1.12-1.14)
Sex discrimination		
No	1 (Reference)	1 (Reference)
Yes	1.22 (1.20-1.24)	1.18 (1.17-1.20)
Being bullied		
No	1 (Reference)	1 (Reference)
Yes	1.16 (1.15-1.17)	1.15 (1.14-1.16)
Deprivation-related ACEs		
Physical neglect		
No	1 (Reference)	1 (Reference)
Yes	1.06 (1.06-1.07)	1.06 (1.05-1.06)
Emotional neglect		
No	1 (Reference)	1 (Reference)
Yes	1.11 (1.11-1.12)	1.10 (1.09-1.11)
Parental divorce		
No	1 (Reference)	1 (Reference)
Yes	1.05 (1.05-1.06)	1.04 (1.03-1.05)
Household criminality		
No	1 (Reference)	1 (Reference)
Yes	1.08 (1.06-1.10)	1.07 (1.04-1.09)
Household mental illness		
No	1 (Reference)	1 (Reference)

© 2024 He Y et al. *JAMA Network Open.*

eTable 11. Associations between each ACE subtype and NSSI (defined as reporting 5 or more times) (continued).

Yes	1.22 (1.19-1.25)	1.20 (1.17-1.23)
Family financial problems		
No	1 (Reference)	1 (Reference)
Yes	1.10 (1.09-1.11)	1.08 (1.07-1.09)
Parental death		
No	1 (Reference)	1 (Reference)
Yes	1.04 (1.02-1.05)	1.02 (1.01-1.04)

Abbreviations: ACE, adverse childhood experience; NSSI, non-suicidal self-injury; PR, prevalence ratio.

[a]Model 1 was adjusted for age, sex, current drinking status, current smoking status, household socioeconomic status, and academic pressure.

[b]Model 2 was adjusted for the variables in Model 1, plus supportive school environments.

© 2024 He Y et al. *JAMA Network Open.*

eTable 12. The interactions between ACEs and supportive school environments with NSSI (defined as reporting 5 or more times).

Variable	PR (95% CI)	
	Model 1[b]	Model 2[c]
NSSI		
ACEs	1.14 (1.13-1.15)	1.13 (1.11-1.14)
supportive school environment	0.98 (0.98-0.98)	0.98 (0.98-0.99)
ACEs* supportive school environment	0.98 (0.98-0.99)	0.98 (0.98-0.99)
Interaction ratio[a]	0.87 (0.86-0.88)	0.88 (0.87-0.90)

Abbreviations: ACEs, adverse childhood experience; NSSI, non-suicidal self-injury, PR, prevalence ratio.

[a] calculated as $PR_{\text{ACE* supportive school environment}} / (PR_{\text{ACEs}} \, PR_{\text{supportive school environment}})$

[b] Model 1 was unadjusted.

[c] Model 2 was adjusted for age, sex, current drinking status, current smoking status, household socioeconomic status, and academic pressure.

© 2024 He Y et al. *JAMA Network Open.*

eTable 13. Associations between cumulative ACEs with NSSI, suicidal ideation, and suicide attempt, further adjusted for depressive symptoms.

Variable	PR (95% CI)		
	NSSI	Suicidal ideation	Suicide attempt
	Model[a]	Model[a]	Model[a]
The cumulative of ACEs (1-unit increase)	1.04 (1.03-1.04)	1.05 (1.05-1.05)	1.03 (1.03-1.03)
Any ACEs			
0	1 (Reference)	1 (Reference)	1 (Reference)
≥1	1.07 (1.06-1.07)	1.10 (1.09-1.11)	1.04 (1.04-1.05)
No. of ACEs			
0	1 (Reference)	1 (Reference)	1 (Reference)
1	1.04 (1.03-1.04)	1.06 (1.05-1.06)	1.02 (1.01-1.02)
2	1.07 (1.06-1.08)	1.10 (1.09-1.11)	1.04 (1.03-1.04)
3	1.13 (1.11-1.14)	1.17 (1.16-1.19)	1.08 (1.07-1.09)
≥4	1.19 (1.18-1.21)	1.26 (1.25-1.28)	1.19 (1.18-1.21)
The cumulative of threat-related ACEs	1.06 (1.06-1.07)	1.07 (1.07-1.07)	1.05 (1.05-1.05)
No. of threat-related ACEs			
0	1 (Reference)	1 (Reference)	1 (Reference)
1	1.08 (1.07-1.09)	1.10 (1.09-1.11)	1.03 (1.03-1.04)
≥2	1.18 (1.17-1.19)	1.21 (1.20-1.22)	1.13 (1.12-1.14)
The cumulative of deprivation-related ACEs	1.02 (1.02-1.03)	1.04 (1.04-1.05)	1.03 (1.03-1.03)
No. of deprivation-related ACEs			
0	1 (Reference)	1 (Reference)	1 (Reference)
1	1.03 (1.02-1.03)	1.05 (1.04-1.05)	1.02 (1.02-1.02)
≥2	1.05 (1.04-1.06)	1.10 (1.09-1.10)	1.07 (1.07-1.08)

Abbreviations: ACE, adverse childhood experience; NSSI, non-suicidal self-injury; PR, prevalence ratio.

[a]Model was adjusted for age, sex, current drinking status, current smoking status, household socioeconomic status, academic pressure, supportive school environment, and depressive symptoms.

© 2024 He Y et al. *JAMA Network Open.*

eTable 14. Associations between each ACE subtype with NSSI, suicidal ideation, and suicide attempt, further adjusted for depressive symptoms.

ACE subtype	PR (95% CI)		
	NSSI	Suicidal ideation	Suicide attempt
	Model[a]	Model[a]	Model[a]
Threaten-related ACEs			
Physical abuse			
No	1 (Reference)	1 (Reference)	1 (Reference)
Yes	1.16 (1.14-1.18)	1.19 (1.17-1.20)	1.15 (1.14-1.17)
Sex abuse			
No	1 (Reference)	1 (Reference)	1 (Reference)
Yes	1.14 (1.12-1.16)	1.13 (1.11-1.14)	1.11 (1.09-1.13)
Emotional abuse			
No	1 (Reference)	1 (Reference)	1 (Reference)
Yes	1.15 (1.14-1.17)	1.21 (1.19-1.22)	1.17 (1.15-1.18)
Household substance abuse			
No	1 (Reference)	1 (Reference)	1 (Reference)
Yes	1.13 (1.10-1.16)	1.15 (1.13-1.18)	1.10 (1.08-1.13)
Witness of community violence			
No	1 (Reference)	1 (Reference)	1 (Reference)
Yes	1.10 (1.08-1.11)	1.15 (1.13-1.18)	1.05 (1.04-1.06)
Household domestic violence			
No	1 (Reference)	1 (Reference)	1 (Reference)
Yes	1.09 (1.08-1.10)	1.12 (1.12-1.13)	1.07 (1.06-1.07)
Sex discrimination			
No	1 (Reference)	1 (Reference)	1 (Reference)
Yes	1.11 (1.10-1.13)	1.12 (1.11-1.14)	1.08 (1.06-1.09)
Being bullied			
No	1 (Reference)	1 (Reference)	1 (Reference)
Yes	1.10 (1.09-1.11)	1.11 (1.10-1.11)	1.06 (1.06-1.07)
Deprivation-related ACEs			
Physical neglect			
No	1 (Reference)	1 (Reference)	1 (Reference)
Yes	1.02 (1.02-1.03)	1.05 (1.05-1.06)	1.04 (1.04-1.05)
Emotional neglect			
No	1 (Reference)	1 (Reference)	1 (Reference)
Yes	1.05 (1.04-1.06)	1.09 (1.08-1.10)	1.07 (1.07-1.08)
Parental divorce			
No	1 (Reference)	1 (Reference)	1 (Reference)
Yes	1.03 (1.02-1.03)	1.06 (1.05-1.07)	1.03 (1.03-1.04)
Household criminality			
No	1 (Reference)	1 (Reference)	1 (Reference)
Yes	1.03 (1.01-1.05)	1.09 (1.07-1.11)	1.05 (1.03-1.07)

© 2024 He Y et al. *JAMA Network Open.*

eTable 14. Associations between each ACE subtype with NSSI, suicidal ideation, and suicide attempt, further adjusted for depressive symptoms. (continued)

Household mental illness			
No	1 (Reference)	1 (Reference)	1 (Reference)
Yes	1.14 (1.12-1.17)	1.13 (1.11-1.16)	1.11 (1.08-1.13)
Family financial problems			
No	1 (Reference)	1 (Reference)	1 (Reference)
Yes	1.05 (1.03-1.06)	1.05 (1.04-1.06)	1.02 (1.01-1.03)
Parental death			
No	1 (Reference)	1 (Reference)	1 (Reference)
Yes	1.01 (1.00-1.03)	1.02 (1.00-1.03)	1.01 (0.99-1.02)

Abbreviations: ACE, adverse childhood experience; NSSI, non-suicidal self-injury; PR, prevalence ratio.

[a]Model was adjusted for age, sex, current drinking status, current smoking status, household socioeconomic status, academic pressure, supportive school environments, and depressive symptoms.

© 2024 He Y et al. *JAMA Network Open.*

eTable 15. Associations between supportive school environments with NSSI, suicidal ideation, and suicide attempt, further adjusted for depressive symptoms.

Variable	PR (95% CI)
	Model [a]
NSSI	
supportive school environment	0.98 (0.98-0.99)
Suicidal ideation	
supportive school environment	0.98 (0.97-0.99)
Suicide attempt	
supportive school environment	0.99 (0.98-0,99)

Abbreviations: NSSI, non-suicidal self-injury; PR, prevalence ratio.

[a] Model was adjusted for age, sex, current drinking status, current smoking status, household socioeconomic status, academic pressure, supportive school environments, and depressive symptoms.

© 2024 He Y et al. *JAMA Network Open.*

eTable 16. The interactions between ACEs and supportive school environments, further adjusted for depressive symptoms.

Variable	PR (95% CI)
	Model [b]
NSSI	
ACE	1.14 (1.13-1.15)
Supportive school environment	0.98 (0.97-0.98)
ACE* supportive school environment	0.99 (0.98-0.99)
Interaction ratio	0.88 (0.87-0.89)
Suicidal ideation	
ACE	1.19 (1.18-1.20)
Supportive school environment	0.97 (0.97-0.98)
ACE* supportive school environment	0.98 (0.98-0.99)
Interaction ratio	0.85 (0.84-0.86)
Suicide attempt	
ACE	1.12 (1.11-1.12)
Supportive school environment	0.99 (0.99-1.00)
ACE* supportive school environment	0.98 (0.97-0.98)
Interaction ratio	0.88 (0.87-0.89)

Abbreviations: ACEs, adverse childhood experience; NSSI, non-suicidal self-injury, PR, prevalence ratio.

[a] calculated as $PR_{ACE^{*} \text{ supportive school environment}} / (PR_{ACEs}\ PR_{\text{supportive school environment}})$

[b] Model was adjusted for age, sex, current drinking status, current smoking status, household socioeconomic status, academic pressure, supportive school environments, and depressive symptoms.

© 2024 He Y et al. *JAMA Network Open.*

eReferences

1. Lai W, Wu H, Yang L, et al. Prevalence of unhealthy behaviors and their associations with non-suicidal self-injury, suicidal ideation and suicide attempt among Chinese adolescents. *Child Adolesc Psychiatry Ment Health.* 2024;18(1):61.

2. Anda RF, Croft JB, Felitti VJ, et al. Adverse childhood experiences and smoking during adolescence and adulthood. *JAMA.* 1999;282(17):1652-1658.

3. Felitti VJ, Anda RF, Nordenberg D, et al. Relationship of childhood abuse and household dysfunction to many of the leading causes of death in adults. The Adverse Childhood Experiences (ACE) Study. *Am J Prev Med.* 1998;14(4):245-258.

4. Pace CS, Muzi S, Rogier G, Meinero LL, Marcenaro S. The Adverse Childhood Experiences – International Questionnaire (ACE-IQ) in community samples around the world: A systematic review (part I). *Child Abuse Negl.* 2022;129:105640.

5. Johnson D, Policelli J, Li M, et al. Associations of Early-Life Threat and Deprivation With Executive Functioning in Childhood and Adolescence: A Systematic Review and Meta-analysis. *JAMA Pediatr.* 2021;175(11):e212511.

6. McLaughlin KA, Sheridan MA, Lambert HK. Childhood adversity and neural development: deprivation and threat as distinct dimensions of early experience. *Neurosci Biobehav Rev.* 2014;47:578-591.

7. Bernstein DP, Stein JA, Newcomb MD, et al. Development and validation of a brief screening version of the Childhood Trauma Questionnaire. *Child Abuse Negl.* 2003;27(2):169-190.

8. Peng C, Cheng J, Rong F, Wang Y, Yu Y. Psychometric properties and normative data of the childhood trauma questionnaire-short form in Chinese adolescents. *Front Psychol.* 2023;14:1130683.

9. Xie P, Wu K, Zheng Y, et al. Prevalence of childhood trauma and correlations between childhood trauma, suicidal ideation, and social support in patients with depression, bipolar disorder, and schizophrenia in southern China. *J Affect Disord.* 2018;228:41-48.

10. Qu D, Wang Y, Zhang Z, et al. Psychometric Properties of the Chinese Version of the Functional Assessment of Self-Mutilation (FASM) in Chinese Clinical Adolescents. *Front Psychiatry.* 2021;12:755857.

11. Wang M, Armour C, Wu Y, Ren F, Zhu X, Yao S. Factor structure of the CES-D and measurement invariance across gender in Mainland Chinese adolescents. *J Clin Psychol.* 2013;69(9):966-979.

12. Tsou T. Robust Poisson regression. *J Stat Plan Inference.* 2006;136(9):3173-3186.

13. Tamhane AR, Westfall AO, Burkholder GA, Cutter GR. Prevalence odds ratio versus prevalence ratio: choice comes with consequences. *Stat Med.* 2016;35(30):5730-5735.

14. Martinez B, Leotti VB, Silva G, Nunes LN, Machado G, Corbellini LG. Odds Ratio or Prevalence Ratio? An Overview of Reported Statistical Methods and Appropriateness of Interpretations in Cross-sectional Studies with Dichotomous Outcomes in Veterinary Medicine. *Front Vet Sci.* 2017;4:193.

15. Zocchetti C, Consonni D, Bertazzi PA. Relationship between prevalence rate ratios and odds ratios in cross-sectional studies. *Int J Epidemiol.* 1997;26(1):220-223.

© 2024 He Y et al. *JAMA Network Open.*
