# Supplementary material for: Adverse Childhood Experiences and Nonsuicidal Self-Injury and Suicidality in Chinese Adolescents
Source: JAMA Netw Open. 2024 Dec 30;7(12):e2452816. doi: 10.1001/jamanetworkopen.2024.52816 (PMC11686413; doi:10.1001/jamanetworkopen.2024.52816)
Supplement: Supplement 2. — Data Sharing Statement [file jamanetwopen-e2452816-s002.pdf]

Data Sharing Statement

He. Adverse Childhood Experiences and Nonsuicidal Self-Injury and Suicidality in Chinese Adolescents. *JAMA Netw Open*. Published online December 30, 2024. doi:10.1001/jamanetworkopen.2024.52816

Data
Data available: No
